# Heterogenous treatment effects: secrets for a reliable treat-to-target trial?

**DOI:** 10.1093/cvr/cvx081

**Published:** 2017-05-17

**Authors:** Tomasz J Guzik

**Affiliations:** 1 Institute of Cardiovascular and Medical Sciences, University of Glasgow, Scotland, UK; 2 Department of Internal and Agricultural Medicine, Jagiellonian University, Collegium Medicum, ul. Skarbowa 1, 31-101, Krakow, Poland


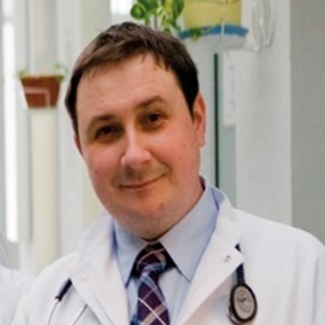


**Commentary on ‘Detecting heterogeneous treatment effects to guide personalized blood pressure treatment: a modeling study of randomized clinical trials’ by Basu *et al.*, *Ann Intern Med*, 2017.^1^**

Over three decades ago Prof Salim Yusuf, Professor Sir Rory Collins and Professor Sir Richard Peto have written the memorable motto to the current clinical research reminding us to ‘always ask important questions and answer them reliably…’.^2^ Although in the era of large randomized clinical trials (RCTs), we believe that we have really understood the key to gaining truly reliable clinical answers, recent clinical trials of hypertension, have however taught us the ‘lessons of humility’.^3^ Over the past year, we have seen an explosion of commentaries and interpretations explaining discrepancies in recent blood pressure target trials including inconclusive ACCORD-BP and SPS3 trials and robustly favorable SPRINT trial.^3^ Simple chance, differences in age, effects of diabetes, or chronic kidney diseases, differences in outcome definitions or BP measurement techniques have all been discussed as causes for discrepancy between results. As clinicians, we are left with a dilemma how to implement these divergent results into our everyday decisions. As clinical researchers, we are left with an important question—how to design a treat-to-target clinical trial that will reliably guide future clinical practice. Finally, as basic researchers—we are facing a dilemma—how to use results of these trials to generate new hypothesis and design experiments, that will have high chance of translation. These are essential questions in cardiovascular research, and recent theoretical modeling approaches using microsimulation provide us with important suggestions.^1^ Treat-to-target trials are very attractive for clinical audiences, because patients are randomized to specific blood pressure goal, which brings them close to clinical situation. Discrepancies between SPRINT and ACCORD conclusions, in spite of their robust designs, have however brought up an important point, that such treat-to-target design may not have sufficient power to examine heterogeneous treatment effects (HTEs), as these occur after randomization.^4^^,^^5^ HTEs encompass differential patient response to specific therapeutic decisions, with some patients responding differently to others. Although classical subgroup analysis is used to address this problem, it does not seem sufficient, as evidenced by comparison of SPRINT and ACCORD trials. Using microsimulation, theoretical modeling, Basu *et al.*^1^ have recently shown that increasing harm at low diastolic BP (<70 mmHg) and diminishing benefit with each additional BP agent, with no benefit >3 agents, may explain differences in the results of SPRINT and ACCORD. Surprisingly, subgroup analysis in these trials failed to show significant interactions among selected pre-specified groups.^6^ Thus, assumptions that, if subgroup analysis of a successful trial fails to show HTEs, then results can be safely generalized to whole trial population may not be correct and lead to mistakes. It is essential to remain very conscious of HTEs and assess the statistical power individual study design has, to detect those. According to Basu *et al.*,^1^ classical treat-to-target design has only 5% statistical power to detect HTEs in spite of very large trial population of >20 000. This is largely associated with the heterogeneity of interventions to achieve target, that is then oversimplified in analysis of aggregate outcomes among intensive treatment and control groups. Although this is a problem in all in treat-to-target trials, such pathophysiological and pharmacological heterogeneity is particularly characteristic for hypertension. In blood pressure treatment trials, we experience within patient heterogeneity and between individual heterogeneity. Differential treatment adherence and response rate is linked to pathophysiological basis of disease heterogeneity including clinical factors baseline CVD risk, blood pressures or co-morbidities and pathophysiologic mechanisms involving central nervous system, vascular, renal, cardiac, and immune components.^7^ Moreover, within-patient heterogeneity with time on individual treatment is also possible and rarely accounted for.^8^ Additional considerations unfold over time of a treat to target study, and would favour individualized sequence of randomized treatments.

## 1. What is the solution?

The Sequential Multiple Assignment Randomized Trial (SMART) research design has been developed explicitly for the purpose of building optimal adaptive interventions by providing answers to clinical questions with complex multifactorial context, as seen for example in hypertension. Indeed, Basu *et al.*^1^ conclude that a trial with sequential randomization to more intensive therapy would have greater than 80% power (as opposed to 5% power in treat-to-target design) even with much smaller sample size. Although the study of Basu *et al.* suffers from some weaknesses and will benefit from additional validation of the model using patient level data, which have now become available from SPRINT, we have to remain very conscious of HTEs when designing a clinical studies in hypertension and when interpreting results. The SMART design of future trials also fits the current need for guiding the development of personalized targeted cardiovascular therapies.^9^^,^^10^ In the case of hypertension, the multi-drug treatment seems to be a major cause of the HTEs; therefore, it is critical to provide a random assignment to addition of each additional agent during intensification of treatment. This also corresponds much more closely to clinical sequential decision-making in the treatment of hypertension depending on the individual heterogeneity in response to treatment.^8^ Although analysis of such trial may be challenging, it is a form of a factorial experimental design, and it allows to truly examine effects of treatment intensification on the way to reaching the blood pressure target.

Thus, considering increasing need of personalized therapies and in pursuit of reliable identification of optimal treatment targets we are likely to use SMART designs in the future and we need to take into account complex heterogenous treatment effects. However, we need to remember that the overarching aim of SMART designs is different to standard RCTs. SMARTs have been created to construct a high-quality adaptive intervention based on data and modeling, while the overarching aim of an RCT is to evaluate an already-existing intervention versus placebo/control treatment.^8^ Increased power to detect heterogeneity of treatment responses may, however, increase scope and usefulness of SMART design for treat-to-target studies in cardiovascular research and in particular in hypertension.

## 2. Implications for a basic scientist

RCTs are a key element of translation of basic findings to clinical practice. We should note, that the results of the study of Basu *et al.*, relate to a specific type of trials—the treat-to-target trials. They however bring our attention to the fact of heterogeneity of clinical responses in such trials, that may reflect limitations in translating basic science findings in RCTs. As we see from microsimulation of SPRINT and ACCORD, in contrast to our basic models, such heterogeneity cannot be easily controlled in simple study designs. Using pure models, in which key variables are constant, is essential for establishment of causal associations and gaining mechanistic insights. The choice of study model is essential for ability to generalize of our basic results to complex clinical settings. A potentially valuable transitional stage could include experiments using fresh human tissues (or organoid cultures) to verify key findings in a more heterogenous, although still controlled, setting. For example, studies in human blood vessels may represent such valuable model which inherently includes HTEs.^11^^,^^12^

Moreover, as most HTEs are pathophysiology based, there is a need to identify the key pathophysiological determinants in basic science experiments, prior to embarking on an expensive and challenging route of RCT translational study. Thus knowledge of pathophysiology is essential for informed design of SMART trial.

Results of this microsimulation are also important to a basic scientist, specifically, within the field of hypertension. In most hypertension-related basic studies we rely on telemetric blood pressure measurement as a major endpoint. The unresolved discrepancy between SPRINT and ACCORD trials makes it questionable, whether this is the best approach in basic studies of novel therapeutic interventions in hypertension. For example, numerous molecules alter vascular function without affecting blood pressure increase,^13^ which in line with ACCORD would be very important and in the light of SPRINT, less essential. Thus understanding heterogenous treatment effects in these trials will help us define in which patients, results of basic studies may be particularly applicable. This can aid better design of next RCTs. It may also help us chose the best study model.

In conclusion, as basic as well as clinican scientists, we should ensure if pathophysiological and pharmacological heterogeneity has been sufficiently accounted for in a trial, if the key HTEs have been identified and what was the power of the design to detect those. It turns out, that simple subgroup analyses presented in the RCTs may not be sensitive enough, and hence a need to seek for analysis of HTEs. Finally, if significant HTEs are seen, which model will most reliably represent those in our ‘from bench to bedside and …back’ studies.

**Conflict of interest:** none declared.

## Funding

This paper was supported by the European Research Council project No 726318, Marie Curie CIG (No. 631773) and British Heart Foundation Centre for Research Excellence (RE/13/5/30177).
